# Linkages between stomatal density and minor leaf vein density across different altitudes and growth forms

**DOI:** 10.3389/fpls.2022.1064344

**Published:** 2022-11-25

**Authors:** Ming Zhang, Huirong Gao, Shuang Chen, Xiaochun Wang, Weiyi Mo, Xue Yang, Xue Wang, Zhibo Wang, Ruili Wang

**Affiliations:** ^1^ College of Forestry, Northwest A&F University, Yangling, Shaanxi, China; ^2^ Institute of Soil and Water Conservation, Northwest A&F University, Yangling, Shaanxi, China; ^3^ Institute of Soil and Water Conservation, Chinese Academy of Sciences and Ministry of Water Resources, Yangling, Shaanxi, China; ^4^ Qinling National Forest Ecosystem Research Station, Yangling, Shaanxi, China

**Keywords:** stomatal density, minor vein density, altitudes, phylogeny, plant growth forms

## Abstract

Water supply and demand in leaves are primarily determined by stomatal density (SD, water demand) and minor leaf vein density (VLA, water supply). Thus, covariation between them is essential for maintaining water balance. However, there is debate over whether these two traits vary in a coordinated way. Here, we gathered SD and VLA data from 194 species over four altitudinal gradients, and investigated their relationships across all species, growth forms, and different altitudes. Our findings demonstrated that SD and VLA were positively associated across all species, independent on plant phylogeny. Moreover, the reliability of this SD-VLA relationship increased with altitudes. Although the stomatal number per minor vein length (SV) remained stable across different altitudes and growth forms, the positive SD-VLA relationship was found only in shrubs and herbs, but not in trees. Differently, a strong coordination between total stomatal number and total leaf vein length was observed across all species, trees, shrubs and herbs. These findings suggested that coordinating stomatal number and minor vein length within one leaf, rather than stomatal and vein density, may be a common choice of plants in the fluctuating environment. Therefore, to explore the relationship between total number of stomata and total length of leaf veins seems to better reflect the linkage between stomata and leaf veins, especially when covering different growth forms.

## Introduction

For plant individuals, stomata regulate the exchange of gas and water vapor between leaf tissue and the atmosphere (Simonin and Roddy, 2018), its variations in shape and distribution generally reflect different adaptive responses to environments (Sun et al., 2021). The evolution of stomata undergone a transformation from large and rare to small and dense (Liu et al., 2019), which is closely associated with decrease in atmospheric carbon dioxide concentration (Feild et al., 2011). Dense stomata contribute to high transpiration (Kassout et al., 2021), whereas water loss is unavoidable as a result of the exchange of water vapor through stomata (Brodribb and Jordan, 2011; Zhao et al., 2020), thus an urgent water supply is needed to compensate for these moisture losses (Scoffoni et al., 2017). Leaf veins transport water for transpiration and photosynthesis and ensure water supply within leaves (Brodribb and Field, 2010; Zhu et al., 2020). Therefore, a necessary linkage of stomata and leaf vein is developed to adapt to changes in hydrological conditions (functional coordination theory) (Brodribb and Jordan, 2011). In fact, water supply and demand in leaves are primarily determined by minor leaf vein density (VLA) and stomatal density (SD), respectively (Brodribb et al., 2017). Currently, many studies have observed a positive relationship between SD and VLA (Carins Murphy et al., 2014; Brodribb et al., 2017; Lei et al., 2018), but negative or marginal correlation is also found in some cases (Zhang et al., 2015; Zhang et al., 2021). Thus, there is still controversy over the direction of SD-VLA coordination and whether such coordination is a common feature.

**Figure 1:**
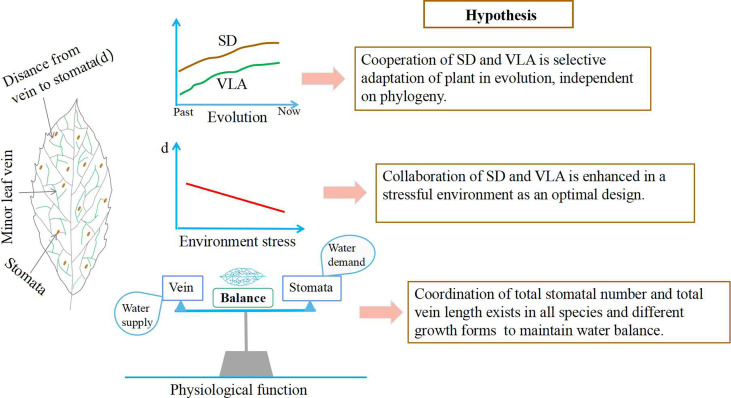
Variation mechanism of the relationship between stomata and leaf veins at different altitudes and growth forms. SD, stomatal density; VLA, vein length per unit area; d, the distance from the end of minor veins to the site of evaporation near the stomata.

At first, evolutionary history of species may have important impact on the covariation of SD and VLA (Brodribb and Feild, 2010; Zhang et al., 2021). Previous studies observed that leaf vein had experienced a dramatic increase during the Cretaceous period (Feild et al., 2011). Similarly, the number of stomata was also increased to maintain photosynthesis due to the decrease in carbon dioxide concentration during the Cretaceous (Brodribb and Field, 2010; Brodribb et al., 2017). It is evident that the increase in both SD and VLA is a plants’ adaptation to the setting of atmospheric changes, and these increases are synchronous and coordinated (Feild et al., 2011). Moreover, Zhang et al. (2014) found that SD and VLA were positively coordinated in a study of eleven species belonging to basal angiosperms whether or not phylogeny is considered. And this result was found in *Paphiopedilum* species (Zhang et al., 2012). These evidences suggest that coordinated change of SD and VLA may be an adaptive strategy of plant in evolution. Therefore, we speculate that SD and VLA are associated across multiple species and that their cooperation as a selective adaption independent on plant phylogeny (Hypothesis 1, **Figure 1**).

Secondly, the coordination of SD and VLA may be influenced by environmental factors. SD and VLA exhibited a significantly positive relationship in adaptation to changes in light (Carins Murphy et al., 2012) and humidity (Carins Murphy et al., 2014). However, some studies have found that SD and VLA respond inconsistently to CO_2_ concentration (Uhl and Mosbrugger, 1999) and vapour pressure difference (Torre et al., 2003). These findings imply that linkage between SD and VLA may be discrepant in different environments. In fact, the correlation of SD and VLA is an optimal design, that is, these two traits are structurally independent, but related because of their common involvement in water regulation within leaves (Sack et al., 2013). However, such trait correlation due to optimal design is indirect and can shift across environments (Sack et al., 2013). In general, plants tend to develop a series of functional traits to increase their fitness in stressful habitats (Mayfield and Levine, 2010). As an optimal design, the coordination of SD and VLA could effectively improve plant photosynthesis and water use efficiency, which benefits plant survival under harsh condition (Brodribb et al., 2017; Lei et al., 2018). Altitudinal gradients can serve as ideal experimental sites to investigate the variation in SD-VLA relationship in different habitats. We hypothesize that the collaboration of SD and VLA may be enhanced in high altitude due to optimal design (Hypothesis 2, **Figure 1**).

Finally, the coordinated change of SD and VLA may be influenced by variation in leaf size, but how the leaf area (LA) controlled the variation in SD, VLA, and their relationship remained unclear (Zhao et al., 2017). For example, Carins Murphy et al. (2012) found that leaf expansion resulted in the coordinated development of SD and VLA in *Toona ciliate*. However, other studies found that minor VLA is little affected by LA (Sack et al., 2012; Zhao et al., 2016), whereas SD and LA revealed different types of associations (Carins Murphy et al., 2014; Alvarez-Maldini et al., 2020). In fact, these differences may be caused by species differences. In general, there are considerable variation in traits such as leaf size, stomata, and leaf vein among growth forms, thus the effects of leaf size to stomatal and leaf vein development may be different (Sack et al., 2013; Liu et al., 2019). As a result, these various effects of LA on SD and VLA may contribute to the mixed results of stomatal and leaf vein coordinated development (Carins Murphy et al., 2012; Zhang et al., 2015; Zhang et al., 2021). However, it is undoubted that the coordination of stomata and veins, which is essential for plant growth, allows for the construction of leaves that maintain balance in water transport and transpirational water loss (functional coordination theory) (Brodribb and Jordan, 2011; Evans-Fitz. Gerald et al., 2016). It thus appears that the SD-VLA relationship could not accurately reflect the general rule of water demand-supply balance in leaves, due to the effect of leaf size. In comparison with stomatal and vein density, the total stomatal number and leaf vein length within one leaf could better reflect the quantity of water demand and supply. Therefore, we expect that total number of stomata and total length of leaf vein within one leaf would covary across all species and different growth forms (Hypothesis 3, **Figure 1**).

To test these hypotheses, we examined SD and VLA of 194 plant species along four altitudinal gradients in central China, which comprised 39 tree species, 53 shrub species, and 102 herb species. This dataset allowed us to test the generality and direction of the SD-VLA relationship across different habitats and plant growth forms. Meanwhile, we explored the relationship between total number of stomata and total length of leaf vein across all species, trees, shrubs and herbs. In addition, we investigated the influence of plant phylogeny and LA on SD-VLA relationship, and compared the strength of SD-VLA coordination in different altitudinal gradients and growth forms. We also used SV (the stomatal number per minor vein length) to examine the coordination between water demand of a given number of stomata and water supply per unit length of minor veins. As a ratio of SD to VLA, a stable SV indicated the water supply-demand balance (Zhao et al., 2017).

## Materials and methods

### Study site and sampling

Leaf samples were gathered on the northern slope of Taibai Mountain Nature Reserve in Shaanxi Province, central China (107°19′—107°58′ E, 33°49′—34°10′ N). The height of nature reserve ranges from 1060 m to 3771 m, with the annual precipitation between 656 mm to 869 mm and an annual average temperature between -2.10°C to 13.27°C (Tang et al., 2004). Topographic and climatic differences cause a vertical zonation of major forest along this height, including deciduous oak forest, birch forest, coniferous forest, and subalpine shrubland-meadows.

In July 2017, field sampling was carried out. Our sampling sites ranged in elevation from 1374 to 3649 m above sea level. We set four altitudinal gradients to sample and each gradient represents a typical vegetation type (**Supplementary Table S1**). In each altitudinal gradient, four experimental plots (20 × 20 m plot for forest sites) were established, with two 5 × 5 m plots for shrubs and five 1×1 m plots for herbs nested in each of forest plot. Three mature and healthy species from each plot were randomly chosen, and 30 to 40 of their fully extended, sun-exposed leaves were sampled (Wang et al., 2020). We collected the leaves of 98, 46, 46, 33 species from four altitudinal gradients, respectively (**Supplementary Table S1**, **S2**). Because some species occur in multiple altitudinal gradients. Finally, 194 species from 137 genera and 70 families were sampled in total (**Supplementary Table S5**), including common and dominant species within the plant communities.

#### Trait measurements

For each species, 5-10 leaves were randomly chosen to measure minor veins density. Each leaf was cut into 1cm^2^ sections (avoiding major vein), and stored in a formalin-acetic acid-alcohol (FAA) fixative. In the laboratory, the 1cm^2^ leaf sample slices were submerged in a 7% (w/v) solution of NaOH (in H_2_O). The solution was replaced every day until the samples were transparent. These samples were then soaked in distilled water for 30 minutes, moved then to 5% (w/v) NaOCl (in H_2_O) for 5 minutes, and rinsed in distilled water for 3 minutes. Next, the leaf samples were stained in 1% (w/v) safranin O for 15 minutes to color the lignin-rich veins. Then, the samples were mounted on microscope slides and photographed at 200× magnification using a Motic BA210 microscope (Motic Medical Diagnostic Systems, Co., Ltd., Xiamen, China). The length of the minor veins was measured manually with Motic Images Plus 3.0 (Motic Medical Diagnostic Systems, Co., Ltd., Xiamen, China). The minor vein density (VLA, mm mm^–2^) was calculated as the total length of minor veins per unit area. For each species, at least three leaves from different individuals were used and 15 fields of view were selected (Sack et al., 2012; Zhang et al., 2015; Wang et al., 2020).

The nail-polish imprint method was used to measure stomatal density (SD) (Wang et al., 2015). The major veins were avoided when clear nail polish was placed on the abaxial leaf surface. After the polish dried, and then we peeled off and mounted the polish onto a glass slide. For each species, at least three leaves from different individuals were selected for stomatal observations. Their stomata were counted in 12 randomly chosen fields. SD (no. mm^-2^) was calculated as the stomata number per unit area. We also calculated the value of SV (no. mm^-1^), i.e., the ratio of SD to VLA (Zhao et al., 2017). Finally, Leaf images were taken using a scanner and leaf area was measured using Image J software (National Institutes of Health, Bethesda, MD, USA). Total stomatal number and total vein length was defined as stomatal density and vein density multiplied by leaf area, respectively.

### Measurement of environmental factors

The mean annual temperature (MAT) and mean annual precipitation (MAP) at these sampling sites in this study were taken from a global database with a spatial resolution of approximately 1 km^2^ (http://www.worldclim.org) (Wang et al., 2020). Aridity index data (AI, precipitation/evapotranspiration) were downloaded from Global raster data (CGIAR-CSI, https://cgiarcsi.community) at 30 arcsec resolution. Low value of AI indicates a drier climate.

The 0-10 cm soil layer was sampled using a soil drill with a 6 cm diameter. In each plot, 30 sampling sites were randomly chosen to collect soil and then mixed. Subsequently, after air-drying and removing any pebbles and plant debris, the soil samples were ground to pass a 2-mm sieve. The soil organic carbon (SOC), total nitrogen (TN), and total phosphorus (TP) were measured by elemental analyzer (Vario MAX CN Elemental Analyzer, Elementar, Germany).

### Species phylogeny

According to the Plant List (http://www.theplantlist.org/), we revised species’ names. The comprehensive species-level angiosperm phylogeny in phylomatic version 3 (http://phylodiversity.net/phylomatic/) was used to construct a phylogenetic tree (Zanne et al., 2014). Species classification was matched to the Angiosperm Phylogeny Group IV classification (APG, 2016).

### Data analysis

Trait data were log10-transformed to meet the assumption of normality when necessary. Data of each species was classified into different growth form, including trees, shrubs, and herbs. To compare the difference in SD, VLA, and SV among three growth forms, a one-way analysis of variance (ANOVA) and least significant difference (LSD) multiple comparisons were carried out. We also compared the difference in SV among different altitudes by using ANOVA. Then, the changes in SD and VLA along altitudinal gradients were examined using regression analyses. Models with the highest coefficient of determination (R^2^) and lowest Akaike information criterion (AIC) values were chosen as the best-fit models for each trait.

To examine the coordination between SD and VLA, the bivariate analyses of SD–VLA relationship was first performed using ordinary least squares (OLS) linear regression. Differences in the slopes and elevations of these relationships among growth forms and different altitudes were evaluated by standardized major axis regression (SMA). The scaling relationship between SD and VLA was described by the equation: *log (y)* = *log (b)* + *a log (x)*, where *y* was SD, and *x* was VLA; *a* was the y-intercept and *b* was the slope of the scaling function. The slope of a linear relationship was subjected to a heterogeneity test at various altitudes and growth forms, and a common slope was determined when these individual slopes were homogeneous (Warton et al., 2012). The differences between the intercepts and the slopes were then compared using multiple comparison tests. These analyses were performed by (S)MATR Version 2.0. Moreover, linear regression models were used to evaluate relationship between total number of stomata and total length of leaf vein across all species, trees, shrubs and herbs. The linear regression was also used to analyze the relationship between SD and VLA with LA in different growth forms.

Blomberg’s *K* statistic was performed to evaluate the conservation of trait development. Significance was testing *via* comparison of the variance of standardized contrasts to random values obtained by shuffling trait data across the tips of the tree 999 times. Greater phylogenetic conservatism for the given characteristic is indicated by higher *K* values (Blomberg et al., 2003). Moreover, to examine the phylogenetic influence on SD-VLA relationships, phylogenetically independent contrasts (PIC) was performed across all species. According to previous studies (Wikström et al., 2001), the divergence time of the family was defined as the divergence time of the earliest genus within a family (Wang et al., 2019). We also utilized regression analyses to investigate the association between three traits and divergence time for each family.

Finally, we used a linear mixed-effects model to examine the effect of environmental factors on SD and VLA. The ‘lmer’ function in the R package ‘lme4’ was then used to build a full model with traits as response variables, environmental variables as fixed effects, and altitude as random effects. Models are simplified using the ‘dredge’ function in package ‘MuMIn’, and Bayesian Information Criterion (BIC) was used to compare models. The smallest BIC value was chosen as the best-fit models. The ‘vif’ function was carried out to calculate the variance inflation factor (VIF) for measuring the collinearity among the explanatory variables, and variables with VIF > 7 were excluded. Marginal R^2^ (R^2^m) denoted the proportion explained by the fixed effect, while conditional R^2^ (R^2^c) denoted the proportion explained by both the fixed and random effects. Finally, the individual contribution of each selected fixed-factor was determined by ‘glmm.hp’ function (Lai et al., 2022).

## Result

### Variation in stomatal and leaf vein traits

Both of stomatal and leaf vein traits showed moderate variation between species (**Table 1**). SD ranged from 32.90 to 669.98 no. mm^-2^ (coefficient of variation, CV=0.59), the VLA ranged from 1.29 to 14.69 mm mm^-2^ (CV=0.38), and the SV ranged from 7.09 to 125.39 no. mm^-1^ (CV=0.56). There were significant differences in leaf vein and stomatal characteristics among different plant growth forms. Trees and shrubs had greater mean SD and VLA than herbs (*P*<0.05, **Table 1**). Nevertheless, SV remained relatively constant among different growth forms (**Table 1**).

**Table 1:** Differences in leaf functional traits among plant growth forms.

Traits	Growth form	N	Max	Min	Mean ± SD	CV
SD	Total	194	669.98	32.9	159.87 ± 94.97	0.59
Stomatal density	Tree	39	669.98	84.38	209.04 ± 128.62a	0.62
(no. mm^-2^)	Shrub	53	614.65	32.9	173.91 ± 100.40a	0.58
	Herb	102	324.52	43.37	133.78 ± 63.99b	0.48
VLA	Total	194	14.59	1.29	5.51 ± 2.12	0.38
Vein length per leaf area	Tree	39	14.59	1.29	6.75 ± 2.53a	0.37
(mm mm^-2^)	Shrub	53	10.48	3.14	6.06 ± 1.90a	0.31
	Herb	102	11.44	1.88	4.76 ± 1.74b	0.37
SV	Total	194	125.39	7.09	30.62 ± 17.06	0.56
The stomatal number per minor vein length	Tree	39	125.39	8.71	35.27 ± 24.64a	0.70
(no. mm^-1^)	Shrub	53	82.08	7.09	30.32 ± 18.05a	0.60
	Herb	102	69.31	11.16	29.00 ± 12.20a	0.42

N, species number; Trait values are mean value ± SD (standard deviation); CV, coefficient of variation. Statistical differences are denoted by different letters (*P* < 0.05).

**Figure 2:**
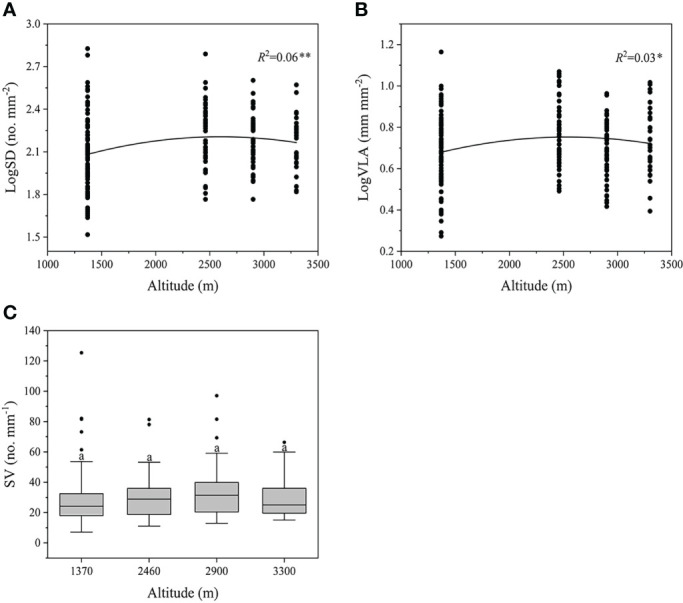
Changes in SD, VLA, and SV across all species along the altitudes. Different letters indicate significant differences (*P*<0.05). SV, stomatal number per minor vein length. **P<0.01, *P<0.05. Trait abbreviations are provided in **Figure 1**.

Along the altitudinal gradients, we found significantly quadratic patterns of SD and VLA (*P*<0.05), where both traits initially increased with altitude and reached a plateau at the middle altitude (**Figure 2A, B**). However, these trends were relatively weak (*R* ^2^ = 0.03-0.06). Differently, SV didn’t exhibit a clear trend with the increased altitude (*P*>0.05, **Figure 2C**).

Additionally, we found that MAT had significant but weak influence on SD (*R* ^2^m=2.00%, *P*<0.05), yet did not significantly affect VLA and SV (**Table 2**). Furthermore, there was no significant effect of TN and TP on SD, VLA and SV, and fixed factors had only small explanatory power for SD (3.00%), VLA (2.51%) and SV (0.75%) (**Table 2**).

**Table 2:** Statistical model for altitude-related variation in stomata and vein trait as a function of mean annual temperature (MAT), total nitrogen (TN), total phosphorus (TP).

	SD				VLA			SV		
	df	Estimate	SS%	*P*	Estimate	SS%	*P*	Estimate	SS%	*P*
Intercept	223	241.32			7.17			40.23		
MAT	223	-4.95	2.00	0.01	-0.09	2.10	0.66	-0.42	0.58	0.56
TN	223				-0.27	0.41	0.80	-1.84	0.17	0.60
TP	223	-94.10	1.00	0.09						
R^2^m			3.00			2.51			0.75	
R^2^c			3.00			19.23			1.93	

df, degree freedom; SS%, percentage of sum of squares explained. Linear mixed-effects model was fit by restricted maximum likelihood. Random effects in model were ‘altitude’. Marginal R^2^ (R^2^m) is concerned with variance explained by fixed factors, and conditional R^2^ (R^2^c) is concerned with variance explained by both fixed and random factors. Trait abbreviations are provided in **Table 1**.

#### Phylogenetic influence on trait variation

According to Blomberg’s *K*, all three traits exhibited significantly phylogenetic signals across all species (**Table 3**). However, the phylogenetic influences differed among plant growth forms. Similar to all species, all of three traits in trees showed significantly phylogenetic signals (*K*=0.16-0.51, all *P*<0.05, **Table 3**). For herbs, significantly phylogenetic signals occurred in both SD and VLA (*K*=0.21-0.35, all *P*<0.05, **Table 3**), but not in SV. However, only SD showed significantly phylogenetic signal in shrubs (*K*=0.33, *P*=0.04, **Table 3**).

**Table 3:** Effects of phylogeny on SD, VLA and SV.

	Total		Tree		Shrub		Herb	
Traits	*K*	*P*	*K*	*P*	*K*	*P*	*K*	*P*
SD	0.26	< 0.01	0.23	< 0.01	0.33	0.04	0.35	< 0.01
VLA	0.38	< 0.01	0.51	< 0.01	0.29	0.17	0.21	0.03
SV	0.14	0.04	0.16	0.02	0.19	0.34	0.18	0.13

Trait abbreviations are provided in **Table 1**.

**Figure 3:**
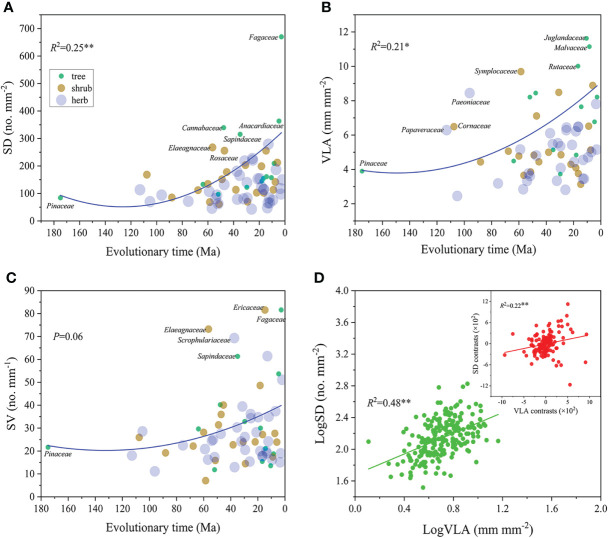
Relationship of SD, VLA and SV with evolution time **(A–C)** and relationship between SD and VLA **(D)**. Circles are weighted by the species number of each family. The larger the circle, the more species it contains. In panel **(D)**, green circles represent the ordinary least squares (OLS) linear regression; red circles represent phylogenetically independent contrast (PIC) correlation. **P<0.01, *P<0.05. Trait abbreviations are provided in **Figure 1**.

Both of SD and VLA showed significantly positive correlations with evolutionary time (*R* ^2^ = 0.21-0.25, *P*<0.05, **Figure 3A, B**), yet SV did not show change significantly with evolutionary time (*P*>0.05, **Figure 3C**). Additionally, SD and VLA showed a significantly positive correlation across all species (*R* ^2^ = 0.48, *P*<0.01, **Figure 3D**). This relationship remained significant even after eliminating phylogenetic effect by PIC analysis (*R* ^2^ = 0.22, *P*<0.01, **Figure 3D**).

#### Covariation between SD and VLA among different altitudes and growth forms

**Figure 4:**
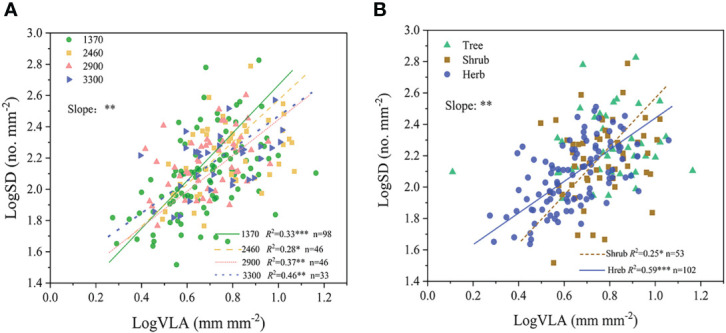
Relationship between SD and VLA at different altitudes **(A)** and growth forms **(B)**. **P<0.01; *P<0.05. Trait abbreviations are provided in **Figure 1**.

Positive relationship between SD and VLA occurred at different altitudes (*R* ^2^ = 0.28-0.46, all *P*<0.05, **Figure 4A**). Moreover, results of SMA showed that the slope of the SD-VLA relationship was affected significantly by altitude (*P <*0.05), with higher slope value in low altitude (**Supplementary Table S4**).

**Figure 5:**
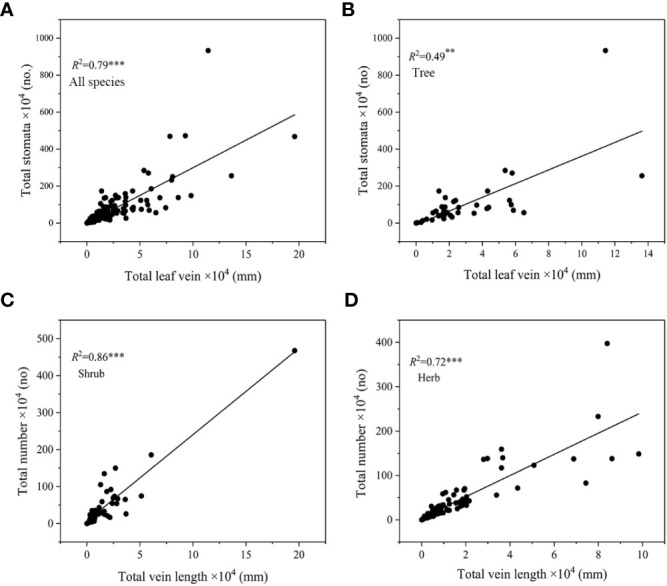
Relationship between total number of stomata and total length of leaf vein in all species **(A)**, trees **(B)**, shrubs **(C)** and herbs **(D)**. **P<0.01, ***P<0.001.

In the case of different growth forms, SD-VLA linkage was found in shrubs and herbs (*R* ^2^ = 0.25-0.59, *P*<0.05, **Figure 4B**), and the slope of SD-VLA relationship of shrubs was higher than that of herbs (*P*<0.05, **Supplementary Table S4**). Different from shrubs and herbs, SD and VLA were decoupled in trees (**Figure 4B**). In addition, a strongly positive relationship of total number of stomata and total length of leaf vein was found across all species, trees, shrubs and herbs (*R* ^2^ = 0.49-0.86, all *P*<0.01, **Figure 5**).

## Discussion

### Correlation between SD and VLA over evolutionary time

How to construct leaves with a balance between water supply and demand is a major problem for plant growth (Carins Murphy et al., 2014; Evans-Fitz. Gerald et al., 2016), the key strategy to address this issue is to coordinate SD and VLA (Brodribb et al., 2017) because they were responsible for water demand and supply in leaves, respectively (Sack and Scoffoni, 2013; McElwain et al., 2016). In our study, positive SD-VLA relationship occurred across all species and was independent on plant phylogeny (**Figure 3D**), which confirmed our first hypothesis. Moreover, SD and VLA showed the consistent trend over the evolutionary time (**Figure 3A, B**), indicating that stomata and leaf veins have undergone the coevolution over the divergence time (Brodribb and Jordan, 2011; Feild et al., 2011). It is well known that the Cretaceous period experiences profound changes in the composition of the atmosphere, including a long-term reduction in CO_2_ and an increase in O_2_ atmospheric concentrations (Feild et al., 2011; McElwain et al., 2016), and both directions of atmospheric change contributed to an increase in the cost of transpiration for carbon uptake (Feild et al., 2011). In fact, this transpirational cost mainly involved in the energy consumption in constructing and maintaining leaf vein. Thus, plants evolved higher VLA in this context of atmosphere change. Meanwhile, the photosynthetic rate inevitably declines due to the reduction in the concentration of CO_2_ in the atmosphere, thus plant has to invest more stomata to absorb CO_2_ to support photosynthesis. In fact, the increase in SD and VLA is not completely independent and is interactive. Plants with high VLA usually developed denser stomata to meet faster water exchange requirements (Jolly et al., 2021); in turn, high SD also was required more leaf veins to make up for transpiration loss (Brodribb et al., 2017). Therefore, SD and VLA co-varied and created a stable connection during long-term evolution, and this connection was not affected by plant phylogeny.

In addition, we found that the increase in SD and VLA clearly began in the middle of the Cretaceous (100–80 Ma) in our study (**Figure 3A, B**), which further revealed that the changing atmospheric composition during the mid to late Cretaceous had a significant impact on leaf hydraulic and physiological structures (Elliott-Kingston et al., 2016). Previous studies have found that water transport efficiency within leaves was largely dependent upon the distance from the end of minor veins to the site of evaporation near the stomata (Sack and Frole, 2006; Brodribb et al., 2017). Thus, from the view of structural design, the water transport distance could be shortened by regulating structural configurations of stomata and leaf veins, which was of great significance in improving efficiency of water transport in leaves (Xiong et al., 2015). Sack and Scoffoni (2013) pointed out that two structurally independent traits could be associated due to their involvement in a common physiological function. Although this correlation was indirect, it existed in many processes. In fact, SD and VLA took advantage of opportunity of changes in atmospheric composition to optimize their structural design thereby developing a closer linkage (Feild et al., 2011; Sack and Scoffoni, 2013). Therefore, as an optimal design, the SD-VLA coordination might be a common strategy adapted by plant to adapt to changing environment.

Furthermore, as the key of water supply and demand, coordinated change of leaf vein and stomata have paramount significance in maintaining water balance in leaves (Brodribb et al., 2017). Brodribb and Jordan (2011) first found that the response of SD and VLA to environmental conditions achieved a homeostatic balance between hydraulic and stomatal conductivities, suggesting the inevitability of collaboration between stomata and leaf vein. In our study, we found that SV did not show significant trend with evolutionary time (**Figure 3C**). This indicates that SV retain stable in evolution, suggesting a balance of water supply and demand and providing further support for the coordination of SD and VLA in long-term evolution. In conclusion, the cooperation of stomata and leaf veins is a selective adaptation developed in plant evolution.

#### Coordination between SD and VLA across different altitudes

Our study showed a statistically positive correlation of SD and VLA at all four altitudinal gradients (*R* ^2^ = 0.28-0.46, all *P*<0.05, **Figure 4A**). In contrast with low altitudes, the higher reliability of SD-VLA relationship occurred in high altitudes (*R* ^2^ = 0.46 vs. 0.28-0.37, **Figure 4A**), which consistent with our second hypothesis. In general, plants modulate multiple functional traits to adapt to environment, but there are differences in how these traits are combined in ecosystems with distinct dominant environmental factors (Goufo et al., 2017; He et al., 2020). Different from previous studies conducted on manipulated experiments (Zhao et al., 2017; Lei et al., 2018), our study examined the SD-VLA linkage in a natural ecosystem and revealed a strongly positive correlation of SD and VLA in different altitudes. These results indicated that the coordination of SD and VLA may be a common choice of plant to adapt to changing environments. In addition, although coordination of SD and VLA occurred in different altitudes, a higher *R* ^2^-values of SD-VLA relationships in high altitudes, indicating a stronger collaboration between SD and VLA. On one hand, generally speaking, plant functional traits exhibit convergent patterns in stressful habitats, thus the coordination between SD and VLA, as an optimal design, is enhanced by convergence in high altitudes (Xu et al., 2018). On other hand, shrubs and herbs become gradually dominant species at high altitudes, thus this stronger SD-VLA correlation in high altitudes may be associated with plant growth forms. Thus, the reliability of SD-VLA relationship may be affected by habitats, in other word, stomata and leaf veins may develop a host of different strategies to adapt to the external environment, and the differences in these strategies are reflected in the reliability of SD-VLA relationship. However, in our study, environmental factors only partially explained attributes (**Table 2**). Therefore, it is uncertain whether the SD-VLA coordination still could be observed in some extremely harsh habitats, such as Tibetan Plateau, and still needed further study

Furthermore, our results showed significant differences in slope of SD-VLA relationship among different altitudinal gradients (*P*<0.01, **Figure 4A**). A higher value of slope denoted faster increase in SD with VLA increasing. In our study, the slope decreased gradually with altitudes (**Supplementary Table 4**). Generally, the most efficient utilization of stomatal and vein investment occurs when water supply to evaporative surfaces near the stomata is just enough to maintain fully stomatal opening (Franks and Beerling, 2009). At low altitudes, faster increase in SD with increased VLA may lead to a result that water supply from leaf vein cannot meet transpiration demand, thus a part of stomata may be forced to close (Carins Murphy et al., 2014). By contrast, slower increase in SD with increased VLA at high altitudes is appropriate as it just matches water supply. This idea is also confirmed by the strong correlation of SD and VLA at high altitudes (**Figure 4A**). Different from change of slope, we found that SV did not significant difference in different altitudes (**Figure 2C**). As a parameter of functional trait eliminates the leaf area effect, SV reflects the coordination between transpiration of a given number of stomata and water supplied per unit length of minor leaf veins (Zhao et al., 2017). Overall, in different environments, plants may have different proportions of stomata and veins per unit leaf area, but the water balance in one leaf is always maintained.

#### Coordination between stomatal number and leaf vein length across different growth forms

Our study found that SD and VLA were significantly positively correlated in shrubs and herbs (*P*<0.01, **Figure 4B**), but not in trees (**Figure 4B**). However, a strong association between total number of stomata and total length of leaf vein was observed across all species, trees, shrubs and herbs (*R* ^2 =^ 0.49-0.86, all *P*<0.01, **Figure 5**), which is consistent with our third hypothesis. By contrast, we can find that the linkage between stomata and leaf vein is weakened and even decoupled when measuring in unit leaf area (*R* ^2^ = 0.49-0.86 and *R* ^2^ = 0.25-0.59), suggesting leaf size indeed affect coordinated development of stomata and vein. In general, plants use specific tissues to irrigate the lamina (leaf veins) and to regulate water loss (stomata), to approach homeostasis in leaf hydration during photosynthesis (Fiorin et al., 2016). A study found that differential leaf expansion can enable hydraulic acclimation to sun and shade (Carins Murphy et al., 2012), in fact, this is because leaf expansion regulate stomatal number and leaf vein length thereby balance water supply and demand, which is also the reason why total stomatal number and total leaf vein length are closely related in one leaf. Thus, when exploring the linkage between stomata and leaf veins in different plant functional groups, it may be more appropriate to explore the relationship between total stomata number and total leaf veins length.

In our study, SD and VLA of trees exhibited a decoupled pattern, which may be largely affected by LA. Prior research had mostly focused on the separate relationship of SD and VLA with LA (Sack et al., 2012; Alvarez-Maldini et al., 2020: Zhao et al., 2017) and it is uncommon for studies to take into account the impact of LA to SD-VLA relationship. Our study showed a significantly positive relationship between SD and LA in trees (*P*<0.05), whereas VLA was unaffected by LA (**Supplementary Figure S1**). Therefore, the discrepancy in SD and VLA regard to LA may result in their disassociation in trees. In fact, the positive correlation between SD and LA of trees may reflect a unique adaptation. He et al. (2020) proposed a trait-trait relationship, which was arose due to the concerted convergence of two traits, that is, each trait contributes independently and is selected to combine because of their advantage in the given environment. As major contributors to photosynthesis, SD and LA are combined to enhance photosynthesis thereby promoting plants’ growth (Zheng and Van Labeke, 2017). This combination alleviates slow plant growth due to cooler ambient temperatures by accumulating photosynthetic yield and promotes competitive ability of tree species (Espina et al., 2018; Shelef et al., 2019), but it also leads to a decoupling of the SD-VLA relationship in trees. Thus, the coordinated pattern of SD and VLA may be modified according to the different needs of plants.

Different from trees, shrubs and herbs not only showed a stronger relationship between total stomata number and total leaf vein length (*R* ^2^ = 0.86 and *R* ^2^ = 0.72, *P*<0.001, **Figure 5C, D** **)**, but also this relationship persisted in stomatal number and vein length per unit leaf area (i.e., SD and VLA, *R* ^2^ = 0.25 and *R* ^2^ = 0.59, **Figure 3B**). Such inconsistent trait correlations among trees, shrubs and herbs may reflect their different adaptive strategies (Liu et al., 2019). A stable linkage between stomata and vein suggests that maintaining water dynamic stability is more important for shrubs and herbs. Different from strategy for rapid growth in trees, shrubs and herbs may prefer to maintain a balance of water supply and demand within the leaves, thus providing a stable growth environment, indicating a conservative strategy (Brodribb and Jordan, 2011; Manivannan et al., 2021). Moreover, SD and VLA of herbs showed a significant coordination in each altitudinal gradient, but insignificant altitudinal trends in both shrubs and trees (**Supplementary Table S3**). This suggests that plant growth forms may affect significantly relationship between SD and VLA. Therefore, the relationship between SD and VLA in different growth forms still needs to be explored in more species.

Additionally, the slope of SD-VAL relationship also showed a significant difference in shrubs and herbs (*P*<0.05, **Figure 4B**). Compared to shrub, a lower value of slope in herb and a stronger SD-VLA coordination may indicate a more adaptable allocation of stomata to leaf vein (**Supplementary Table S4**). Unexpectedly, although SD-VLA relationship depended on growth forms, SV remained relatively constant among different growth forms (**Table 1**). This is perhaps for this reason that SV as a function parameter eliminates the impact of leaf area (Zhao et al., 2017). Thus, SV could offer a new perspective to examine the SD-VLA relationship in different plant groups.

## Conclusion

Through examining the variation in SD and VLA from 194 species along a 2000-m altitudinal gradient, we observed a significantly positive relationship between SD and VLA across all species, independent on plant phylogeny. And such relationship also occurred in different altitudinal gradients. Moreover, a strong coordination between total stomatal number and total leaf vein length was observed across different growth forms. However, the cooperation between SD and VLA persist only in shrubs and herbs, but not in trees. These results indicated that coordinating stomatal number and leaf vein length may be a common choice for plant to adapt to environmental change. Furthermore, when considering different growth forms, studying the relationship between total number of stomata and total length of leaf veins seems to better reflect the linkage between stomata and leaf veins in plant growth. Overall, our study provides new support for the covariation between stomatal number and leaf vein length from both evolutionary and ecological perspectives, as well as advances our knowledge of plants’ adaptive strategies to different habitats.

## Data availability statement

The original contributions presented in the study are included in the article/**Supplementary Material**. Further inquiries can be directed to the corresponding author.

## Author contributions

MZ and RW conceived the ideas and designed the methodology. WM, HG, SC, XW, ZW and XY collected the data. MZ and XW analyzed the data. MZ wrote the manuscript. All authors contributed to the article and approved the submitted version.

## Funding

This work was partially supported by the National Natural Science Foundation of China (No. 32271611), Youth Talent Support Project of Science and Technology Association in Shaanxi Province (20200203) and China Postdoctoral Science Foundation (2018T111101).

## Acknowledgments

We thank the Shaanxi Taibaishan National Nature Reserve Authority for the authorized access to Taibai Mountain.

## Conflict of interest

The authors declare that the research was conducted in the absence of any commercial or financial relationships that could be construed as a potential conflict of interest.

## Publisher’s note

All claims expressed in this article are solely those of the authors and do not necessarily represent those of their affiliated organizations, or those of the publisher, the editors and the reviewers. Any product that may be evaluated in this article, or claim that may be made by its manufacturer, is not guaranteed or endorsed by the publisher.
