# Supplementary material for: Linkages between stomatal density and minor leaf vein density across different altitudes and growth forms
Source: Front Plant Sci. 2022 Nov 25;13:1064344. doi: 10.3389/fpls.2022.1064344 (PMC9765094; doi:10.3389/fpls.2022.1064344)
Supplement: Supplementary file 1 [file DataSheet_1.docx]

**Tables**

| Vegetation zone | Altitude/m | Latitude  (°N) | Longitude  (°E) | Annual mean temperature /℃ | Annual mean  Precipitation /mm | Number | Dominant species |
| --- | --- | --- | --- | --- | --- | --- | --- |
| Deciduous *oak* forest | 1370 | 34.09 | 107.71 | 13.27 | 656 | 98 | *Quercus wutaishanica、Quercus variabilis* |
| *Birch* forest | 2460 | 34.01 | 107.82 | 3.29 | 789 | 46 | *Betula utilis、Betula albosinensis* |
| coniferous forests | 2900 | 34.00 | 107.81 | 1.49 | 820 | 46 | *Abies fargesii、Larix chinensis Beissn* |
| subalpine shrubland-meadows | 3300 | 33.96 | 107.80 | -1.02 | 862 | 33 | *Salix cupularis、Polygonum macrophyllum* |

**Supplementary Table S1.** Information of sampling sites.

| Altitudes/m | 1370 | 2460 | 2900 | 3300 |
| --- | --- | --- | --- | --- |
| Total | 98 | 46 | 46 | 33 |
| Tree | 30 | 9 | 4 | 0 |
| Shrub | 30 | 16 | 13 | 4 |
| Herb | 38 | 21 | 29 | 29 |

**Supplementary Table S2.** Number of samples for different altitude gradients.

**Supplementary Table S3.** Pearson correlation of SD and VLA of trees, shrubs and herbs at different altitudes.

| SD-VLA | Tree | Shrub | Herb |
| --- | --- | --- | --- |
| 1370 | *P*=0.85 | *P*=0.32 | 0.64** |
| 2460 | *P*=0.79 | *P*=0.15 | 0.45* |
| 2900 | *P*=0.42 | *P*=0.46 | 0.60** |
| 3300 |  | *P*=0.94 | 0.41* |

SD, stomatal density (no. mm^-2^), VLA, vein length per unit area (mm mm^-2^). *, *P*<0.05, **, *P*<0.01.

**Supplementary Table S4**. Differences in the slopes and intercepts of relationship between SD and VLA at different altitudes and growth forms

|  |  | Slope | Intercept |
| --- | --- | --- | --- |
| Altitudes (m) | 1300 | 1.53 | 1.04 |
|  | 2460 | 1.34 | 1.17 |
|  | 2900 | 1.15 | 1.39 |
|  | 3000 | 1.10 | 1.36 |
| Growth forms | shrub | 1.84 | 0.77 |
|  | herb | 1.33 | 1.21 |

**Supplementary Table S5.** Data of SD, VLA and LA for 194 species. SD, stomatal density (no. mm^-2^), VLA, leaf vein density (mm mm^-2^), LA, leaf area, (cm^2^).

| No | **Species** | SD | VLA | LA |
| --- | --- | --- | --- | --- |
| 1 | *Abies fargesii* | 124.87 | 1.29 | 0.34 |
| 2 | *Acer caesium* | 293.12 | 5.71 | 41.79 |
| 3 | *Acer cappadocicum* | 282.65 | 6.06 | 95.76 |
| 4 | *Acer davidii* | 317.79 | 4.68 | 35.54 |
| 5 | *Acer pictum* | 234.79 | 5.89 | 28.47 |
| 6 | *Acer robustum* | 278.16 | 5.41 | 41.83 |
| 7 | *Acer sterculiaceum* | 194.41 | 3.89 | 44.81 |
| 8 | *Acer tataricum* | 602.68 | 4.81 | 28.76 |
| 9 | *Aconitum henryi* | 80.76 | 4.13 | 22.02 |
| 10 | *Aconitum pendulum* | 168.99 | 5.64 | 2.42 |
| 11 | *Aconitum taipeicum* | 163.01 | 4.53 | 19.52 |
| 12 | *Actaea asiatica* | 50.85 | 2.82 | 42.28 |
| 13 | *Actaea cimicifuga* | 70.29 | 4.07 | 30.77 |
| 14 | *Adiantum capillus-veneris* | 106.93 | 3.29 | 3.18 |
| 15 | *Agrimonia pilosa* | 151.04 | 7.17 | 15.80 |
| 16 | *Ajuga ciliata* | 128.61 | 6.47 | 33.24 |
| 17 | *Akebia trifoliata* | 128.61 | 5.51 | 20.26 |
| 18 | *Allium cyaneum* | 83.75 | 4.08 | 18.11 |
| 19 | *Amelanchier sinica* | 157.03 | 8.08 | 20.43 |
| 20 | *Amelanchier sinica* | 183.70 | 5.47 | 4.64 |
| 21 | *Anaphalis aureopunctata* | 134.59 | 4.81 | 17.44 |
| 22 | *Anemone taipaiensis* | 67.30 | 3.69 | 15.67 |
| 23 | *Angelica dahurica* | 177.22 | 5.46 | 14.21 |
| 24 | *Aquilegia ecalcarata* | 194.41 | 6.36 | 5.77 |
| 25 | *Aquilegia yabeana* | 122.63 | 3.49 | 19.21 |
| 26 | *Asarum sieboldii* | 71.78 | 3.19 | 54.51 |
| 27 | *Astilbe rubra* | 115.15 | 6.48 | 28.80 |
| 28 | *Berberis amurensis* | 151.79 | 4.83 | 15.18 |
| 29 | *Betula albosinensis* | 118.14 | 7.85 | 27.66 |
| 30 | *Betula utilis* | 202.64 | 7.87 | 28.08 |
| 31 | *Buckleya lanceolate* | 103.19 | 4.30 | 11.72 |
| 32 | *Buddleja albiflora* | 276.67 | 9.04 | 67.15 |
| 33 | *Bupleurum dielsianum* | 218.34 | 5.53 | 8.71 |
| 34 | *Campanula punctata* | 94.22 | 3.76 | 49.17 |
| 35 | *Campylotropis macrocarpa* | 152.54 | 7.24 | 19.27 |
| 36 | *Cardamine hirsuta* | 135.72 | 4.89 | 13.94 |
| 37 | *Cardamine leucantha* | 306.58 | 5.40 | 20.18 |
| 38 | *Carex capilliformis* | 139.08 | 5.23 | 2.79 |
| 39 | *Carex taipaishanica* | 150.80 | 5.20 | 3.28 |
| 40 | *Carpesium cernuum* | 89.73 | 3.08 | 259.62 |
| 41 | *Carpinus cordata* | 121.13 | 7.00 | 13.85 |
| 42 | *Castanea mollissima* | 346.95 | 6.56 | 82.02 |
| 43 | *Celastrus orbiculatus* | 136.09 | 7.56 | 47.91 |
| 44 | *Celtis sinensis* | 339.48 | 8.45 | 51.08 |
| 45 | *Chloranthus japonicus* | 62.81 | 3.62 | 48.44 |
| 46 | *Chrysanthemum indicum* | 154.04 | 5.53 | 15.16 |
| 47 | *Circaeaster agrestis* | 79.26 | 6.11 | 0.74 |
| 48 | *Cirsium leo* | 284.14 | 7.35 | 19.83 |
| 49 | *Clematis florida* | 107.68 | 3.71 | 15.67 |
| 50 | *Clerodendrum trichotomum* | 142.07 | 5.13 | 48.37 |
| 51 | *Coluria longifolia* | 182.45 | 6.90 | 9.52 |
| 52 | *Coniogramme japonica* | 71.28 | 2.91 | 23.90 |
| 53 | *Convallaria majalis* | 65.80 | 1.88 | 102.12 |
| 54 | *Cornus alba* | 269.19 | 5.85 | 32.14 |
| 55 | *Cornus hemsleyi* | 192.92 | 8.37 | 34.80 |
| 56 | *Cornus controversa* | 124.13 | 6.41 | 68.32 |
| 57 | *Cornus kousa* | 88.23 | 5.34 | 31.04 |
| 58 | *Corydalis edulis* | 115.15 | 2.76 | 6.71 |
| 59 | *Corydalis latiflora* | 179.46 | 6.90 | 9.62 |
| 60 | *Corydalis linarioides* | 164.50 | 2.48 | 4.98 |
| 61 | *Corydalis taipaishanica* | 91.22 | 3.56 | 5.50 |
| 62 | *Crataegus hupehensis* | 157.03 | 6.16 | 27.35 |
| 63 | *Crataegus pinnatifida* | 128.61 | 4.29 | 31.53 |
| 64 | *Cryptotaenia japonica* | 130.11 | 3.74 | 8.85 |
| 65 | *Daphne giraldii* | 113.66 | 6.52 | 7.51 |
| 66 | *Decaisnea insignis* | 85.24 | 4.44 | 63.44 |
| 67 | *Delphinium grandiflorum* | 86.74 | 4.67 | 15.99 |
| 68 | *Deutzia taibaiensis* | 166.00 | 4.33 | 19.27 |
| 69 | *Dianthus caryophyllus* | 44.86 | 1.95 | 22.45 |
| 70 | *Dianthus chinensis* | 157.03 | 8.35 | 0.43 |
| 71 | *Dioscorea nipponica* | 70.29 | 2.46 | 51.20 |
| 72 | *Diospyros lotus* | 97.21 | 8.21 | 71.56 |
| 73 | *Dryopteris setosa* | 43.37 | 2.82 | 18.72 |
| 74 | *Elaeagnus umbellata* | 267.69 | 3.66 | 13.09 |
| 75 | *Eleutherococcus giraldii* | 114.40 | 5.69 | 28.70 |
| 76 | *Eleutherococcus setchuenensis* | 92.72 | 3.45 | 31.98 |
| 77 | *Elymus tsukushiensis* | 97.95 | 5.16 | 6.37 |
| 78 | *Epilobium angustifolium* | 227.31 | 6.29 | 31.01 |
| 79 | *Euonymus alatus* | 134.59 | 6.23 | 8.33 |
| 80 | *Euonymus phellomanus* | 89.73 | 3.90 | 13.95 |
| 81 | *Fraxinus insularis* | 124.13 | 3.77 | 16.81 |
| 82 | *Fraxinus stylosa* | 166.00 | 5.91 | 23.17 |
| 83 | *Gagea serotina* | 65.80 | 3.46 | 1.94 |
| 84 | *Gentiana apiata* | 173.48 | 4.44 | 0.41 |
| 85 | *Gentiana scabra* | 106.18 | 6.26 | 4.10 |
| 86 | *Halenia corniculata* | 83.75 | 2.72 | 2.26 |
| 87 | *Heracleum hemsleyanum* | 197.40 | 4.98 | 15.52 |
| 88 | *Isodon amethystoides* | 86.74 | 5.25 | 82.75 |
| 89 | *Juglans mandshurica* | 127.12 | 14.59 | 44.58 |
| 90 | *Juglans regia* | 188.43 | 8.66 | 65.10 |
| 91 | *Juniperus pingii* | 199.20 | 4.10 | 0.08 |
| 92 | *Kalimeris hispida* | 242.27 | 4.88 | 10.54 |
| 93 | *Kalopanax septemlobus* | 122.63 | 3.73 | 68.91 |
| 94 | *Kerria japonica* | 207.87 | 5.55 | 20.77 |
| 95 | *Lactuca sativa* | 80.76 | 3.34 | 152.18 |
| 96 | *Larix potaninii* | 84.38 | 3.90 | 0.16 |
| 97 | *Ligularia fischeri* | 118.14 | 7.81 | 125.75 |
| 98 | *Ligularia sibirica* | 59.82 | 3.74 | 230.58 |
| 99 | *Lindera obtusiloba* | 173.48 | 6.51 | 56.15 |
| 100 | *Lithospermum zollingeri* | 149.55 | 2.93 | 9.43 |
| 101 | *Litsea pungens* | 133.10 | 4.49 | 35.47 |
| 102 | *Lonicera fragrantissima* | 387.33 | 4.72 | 34.80 |
| 103 | *Lonicera hispida* | 230.06 | 6.02 | 11.49 |
| 104 | *Lonicera stephanocarpa* | 274.42 | 5.79 | 12.96 |
| 105 | *Lonicera tangutica* | 124.13 | 9.15 | 2.51 |
| 106 | *Lonicera tragophylla* | 100.20 | 6.64 | 24.93 |
| 107 | *Lycopodiastrum casuarinoides* | 58.32 | 4.72 | 0.05 |
| 108 | *Meconopsis quintuplinervia* | 173.48 | 4.32 | 16.27 |
| 109 | *Melilotus albus* | 198.90 | 11.44 | 2.10 |
| 110 | *Morus australis* | 212.36 | 8.89 | 220.31 |
| 111 | *Notopterygium incisum* | 275.17 | 5.66 | 49.54 |
| 112 | *Ophiopogon japonicus* | 119.64 | 4.33 | 4.21 |
| 113 | *Oplismenus undulatifolius* | 61.32 | 3.35 | 6.79 |
| 114 | *Padus racemosa* | 128.61 | 5.69 | 33.19 |
| 115 | *Paeonia anomala* | 94.22 | 8.45 | 88.02 |
| 116 | *Papaver nudicaule* | 113.66 | 6.29 | 5.64 |
| 117 | *Parasenecio hastatus* | 70.29 | 4.27 | 79.40 |
| 118 | *Parasenecio pilgerianus* | 95.71 | 2.51 | 146.21 |
| 119 | *Paris polyphylla* | 47.86 | 2.40 | 9.92 |
| 120 | *Parthenocissus laetevirens* | 70.29 | 4.85 | 105.97 |
| 121 | *Pedicularis sp.* | 260.22 | 6.52 | 11.96 |
| 122 | *Pedicularis sylvatica* | 182.45 | 6.36 | 12.01 |
| 123 | *Pertya sinensis* | 212.36 | 6.18 | 6.72 |
| 124 | *Philadelphus incanus* | 102.44 | 3.77 | 22.54 |
| 125 | *Phlomoides megalantha* | 207.87 | 4.87 | 32.35 |
| 126 | *Phlomoides umbrosa* | 224.32 | 4.84 | 61.59 |
| 127 | *Picris hieracioides* | 234.79 | 7.25 | 49.89 |
| 128 | *Pleurospermum cristatum* | 170.49 | 6.94 | 22.34 |
| 129 | *Pleurospermum giraldii* | 187.68 | 8.33 | 0.47 |
| 130 | *Polygonatum odoratum* | 64.31 | 2.22 | 17.15 |
| 131 | *Polygonatum sibiricum* | 137.58 | 3.51 | 8.00 |
| 132 | *Polygonum macrophyllum* | 243.02 | 7.04 | 13.82 |
| 133 | *Polygonum pinetorum* | 94.22 | 3.43 | 21.26 |
| 134 | *Polygonum sp.* | 83.75 | 4.38 | 10.65 |
| 135 | *Populus tremula* | 192.92 | 10.50 | 40.71 |
| 136 | *Potentilla glabra* | 164.50 | 9.26 | 0.61 |
| 137 | *Primula handeliana* | 104.68 | 3.71 | 20.76 |
| 138 | *Primula matthioli* | 58.32 | 2.95 | 15.85 |
| 139 | *Prunus tomentosa* | 164.50 | 7.21 | 33.17 |
| 140 | *Prunus pseudocerasus* | 136.09 | 4.42 | 20.18 |
| 141 | *Pyrola calliantha* | 255.73 | 3.14 | 9.28 |
| 142 | *Quercus aliena* | 669.98 | 8.21 | 139.25 |
| 143 | *Rhamnus davurica* | 113.66 | 5.00 | 8.85 |
| 144 | *Rhodiola dumulosa* | 71.78 | 2.86 | 0.06 |
| 145 | *Rhodiola kirilowii* | 183.95 | 3.92 | 1.95 |
| 146 | *Rhodiola rosea* | 128.61 | 3.94 | 1.93 |
| 147 | *Rhododendron clementinae* | 400.79 | 7.21 | 37.40 |
| 148 | *Rhododendron concinnum* | 189.93 | 4.81 | 10.03 |
| 149 | *Rhus potaninii* | 363.40 | 6.77 | 17.80 |
| 150 | *Ribes giraldii* | 186.94 | 4.60 | 49.30 |
| 151 | *Ribes glaciale* | 187.31 | 4.35 | 6.14 |
| 152 | *Rodgersia aesculifolia* | 145.06 | 3.30 | 109.74 |
| 153 | *Rosa omeiensis* | 121.13 | 9.51 | 0.84 |
| 154 | *Rosa tsinglingensis* | 110.67 | 5.05 | 9.14 |
| 155 | *Rubia cordifolia* | 46.36 | 2.85 | 34.21 |
| 156 | *Rubus flosculosus* | 192.17 | 6.83 | 36.77 |
| 157 | *Salix cupularis* | 130.11 | 6.65 | 11.13 |
| 158 | *Salix taipaiensis* | 200.40 | 10.17 | 2.37 |
| 159 | *Salix babylonica* | 352.94 | 10.47 | 3.75 |
| 160 | *Sambucus williamsii* | 49.35 | 4.56 | 42.64 |
| 161 | *Sanicula chinensis* | 131.60 | 3.54 | 34.50 |
| 162 | *Saussurea baroniana* | 115.15 | 7.40 | 21.84 |
| 163 | *Saussurea japonica* | 128.61 | 4.14 | 21.56 |
| 164 | *Saussurea odontolepis* | 179.46 | 7.04 | 28.88 |
| 165 | *Saussurea purpurascens* | 157.03 | 8.34 | 10.02 |
| 166 | *Saussurea tsinlingensis* | 198.90 | 4.20 | 199.83 |
| 167 | *Schisandra sphenanthera* | 46.36 | 5.99 | 36.27 |
| 168 | *Scrophularia sp.* | 180.95 | 2.61 | 16.89 |
| 169 | *Sedum aizoon* | 154.04 | 4.43 | 0.79 |
| 170 | *Silene baccifera* | 83.75 | 3.46 | 23.54 |
| 171 | *Sinosenecio oldhamianus* | 83.75 | 4.99 | 11.12 |
| 172 | *Smilax stans* | 32.90 | 3.58 | 12.43 |
| 173 | *Smilax riparia* | 89.73 | 4.13 | 47.46 |
| 174 | *Sorbaria sorbifolia* | 614.65 | 7.55 | 17.13 |
| 175 | *Sorbus tianschanica* | 201.89 | 5.70 | 11.56 |
| 176 | *Sorbus alnifolia* | 88.23 | 6.23 | 26.92 |
| 177 | *Sorbus discolor* | 177.96 | 7.59 | 1.00 |
| 178 | *Sorbus folgneri* | 204.88 | 3.72 | 27.32 |
| 179 | *Sorbus sp.* | 124.13 | 8.19 | 42.74 |
| 180 | *Spiraea alpina* | 271.35 | 10.48 | 2.20 |
| 181 | *Spiraea japonica* | 269.19 | 6.50 | 21.98 |
| 182 | *Spiraea salicifolia* | 186.94 | 6.91 | 6.75 |
| 183 | *Stellaria aquatica* | 324.52 | 5.49 | 2.32 |
| 184 | *Symplocos paniculata* | 68.79 | 9.70 | 37.99 |
| 185 | *Thladiantha dubia* | 76.27 | 4.70 | 43.21 |
| 186 | *Tilia chinensis* | 209.37 | 11.14 | 122.30 |
| 187 | *Toxicodendron vernicifluum* | 157.03 | 9.07 | 63.26 |
| 188 | *Triosteum pinnatifidum* | 124.13 | 6.22 | 110.67 |
| 189 | *Viburnum dilatatum* | 134.59 | 4.78 | 54.53 |
| 190 | *Vicia gigantea* | 279.66 | 4.55 | 20.95 |
| 191 | *Viola acuminata* | 91.22 | 5.14 | 17.41 |
| 192 | *Viola arcuata* | 124.13 | 5.19 | 12.04 |
| 193 | *Vitis piasezkii* | 49.35 | 5.32 | 13.11 |
| 194 | *Zanthoxylum bungeanum* | 154.78 | 10.01 | 3.04 |

**
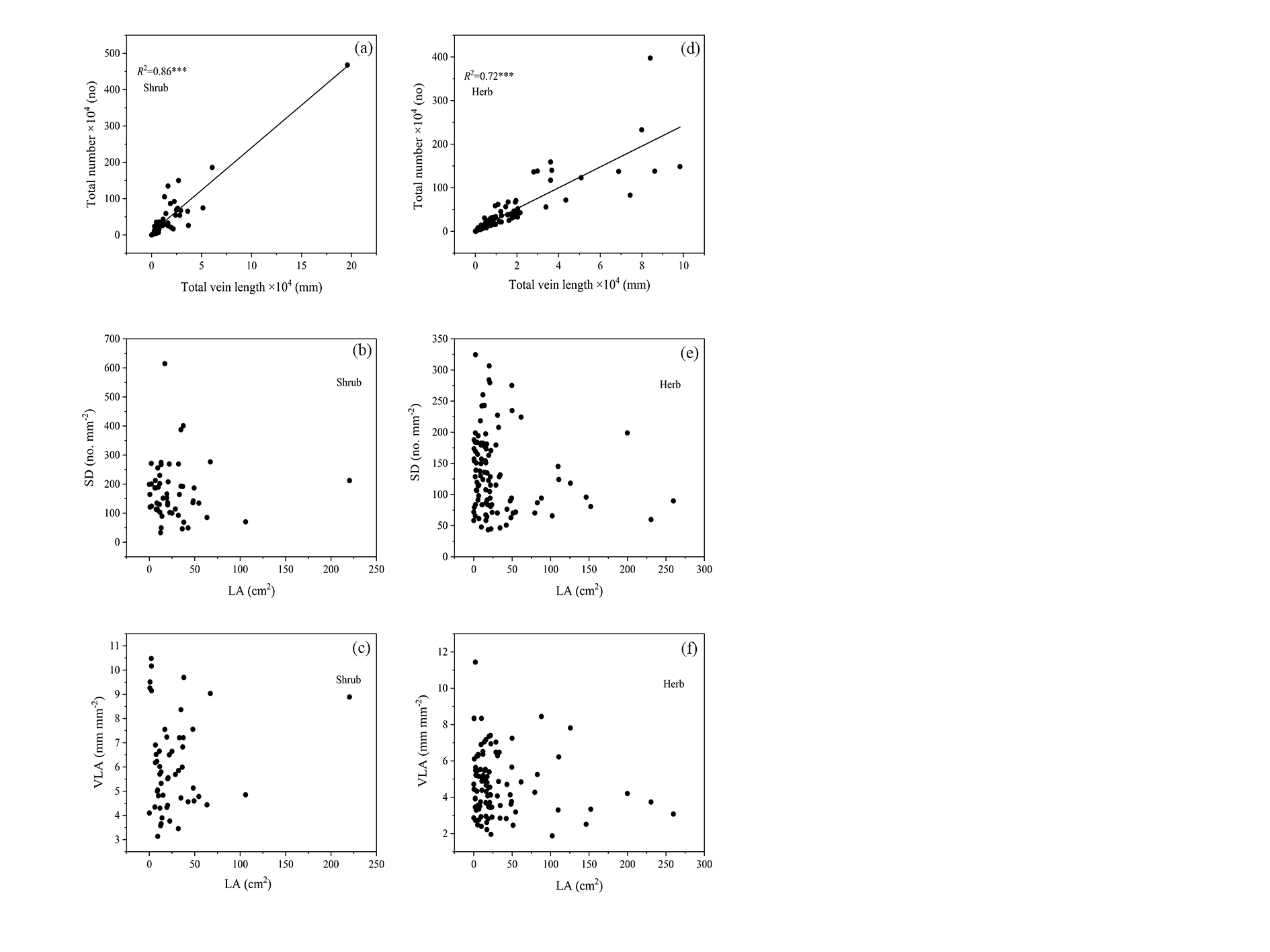
Figure**

**Supplementary Figure S1**. The relationship between total number of stomata and total length of leaf vein in shrubs (a) and herbs (d) and the relationship of SD and VLA with LA in shrubs (b-c) and herbs (e-f). SD, stomatal density, VLA, leaf vein density, LA, leaf area.
